# Capsaicin-Assessed Cough Reflex in Asthma Patients

**DOI:** 10.33549/physiolres.935540

**Published:** 2025-06-01

**Authors:** Martina NEUSCHLOVA, Peter KUNC, Renata PECOVA

**Affiliations:** 1 Department of Pathological Physiology, Comenius University in Bratislava, Jessenius Faculty of Medicine in Martin, Slovak Republic; 2 Clinic of Pediatric Respiratory Diseases and Tuberculosis, National Institute of Pediatric Tuberculosis and Respiratory Diseases, Dolny Smokovec, Comenius University in Bratislava, Jessenius Faculty of Medicine in Martin, Slovak Republic

**Keywords:** Cough, Cough reflex sensitivity, Capsaicin, Asthma

## Abstract

The cough reflex is a primary defensive mechanism for airway protection. Cough disorders are characterized by a change in the threshold for initiating the cough reflex. Various afferent inputs influence the onset and sensitivity of the cough reflex. The study aimed to determine and compare the cough reflex sensitivity between adults with asthma and healthy adults with no history of allergic, respiratory, cardiovascular, gastrointestinal, or endocrine diseases, using European Respiratory Society guidelines on the assessment of cough. We created a group of 52 healthy volunteers (mean age 33.8 years) to serve as a control group for future research into the cough reflex in defined patient groups in upcoming clinical-physiological studies. We found that in the group of healthy volunteers, the threshold concentration of capsaicin required to elicit at least two coughs (C2) was 8.46 μmol/l, while the threshold concentration for inducing at least five coughs (C5) was 26.62 μmol/l. We then compared the reactivity of afferent nerve endings in the airways of healthy adults and adults with asthma by evaluating the differences in the concentrations of capsaicin that elicited C2 and C5 in these two groups. In a group of 19 asthmatic adults (mean age 33 years), the threshold concentration of capsaicin required to induce C2 was 2.03 μmol/l, while the threshold concentration for C5 was 3.02 μmol/l. We demonstrated a significant increase in cough reflex sensitivity in adults with asthma, expressed as the C2 capsaicin concentration (P<0.01) and the C5 concentration (P<0.001).

## Introduction

Cough is a vital protective reflex that prevents aspiration and enhances airway clearance [1]. It protects the airways and lungs from inhaled irritants, particulates, pathogens and clears accumulated secretions from the air spaces [2]. However, pathologically excessive and persistent cough is a common and disabling problem, affecting approximately 5 to 10 % of the adult population [3]. Cough sensitivity is defined as the intensity of the cough reflex in response to various stimuli. Cough can be elicited by the activation of chemically and mechanically sensitive vagal afferent nerves [1,2,4,5]. The cough reflex is a neuronally mediated pathway in which afferent C- and Aδ-fibres innervating airway epithelial and subepithelial cells, express ion channels activated by various stimuli, including capsaicin (chilli pepper extract) [4,6].

Cough may be triggered by an excessive stimulation of the normal cough reflex, such as after the inhalation of foreign bodies or noxious vapors [1,7]. Noxious stimuli are detected by the receptors and ion channels (e.g. TRPV1, TRPA1) localized in the vagal afferent nerve endings of the airway mucosa [8]. Most patients with chronic cough exhibit cough reflex hypersensitivity, responding to low levels of thermal, chemical, or mechanical stimuli [1,7]. Capsaicin is a cough challenge agent that activates the transient receptor potential vanilloid 1 (TRPV1) ion channels, which are primarily located on unmyelinated C-fibers [6,9–11]. Agonists of TRPV1 capsaicin receptors are amongst the most potent chemical stimuli which cause cough [12]. Capsaicin is the most commonly used non-acid tussive agent to experimentally induce cough in humans [13,14]. Cough receptors and C-fibers may interact centrally with afferent pathways innervating receptive fields from the larynx, conducting airways, lungs, pharynx and esophagus, contributing to the increased cough sensitivity in chronic diseases such as asthma, upper airways disorders (UACS) and gastroesophageal reflux disease (GERC) [1,15].

A limitation of measuring cough reflex sensitivity is the wide variability in the methods or equipment currently used for capsaicin challenges [16]. European Respiratory Society (ERS) published standardized inhalation cough challenge methodology to enable consistent interpretation and comparison of data generated by different laboratories [14].

The aim of our study was to evaluate differences of cough reflex sensitivity between healthy adults and asthmatic adults, using ERS guidelines on the assessment of cough [14].

## Methods

This study was designed in line with the ethical principles of the Declaration of Helsinki (1975, revised 2013), the procedure was approved by the Ethics Committee of Jessenius Faculty of Medicine in Martin, Slovakia and the Ethics Committee of Žilina Self-Governing Region, Slovakia. Informed consent was obtained from all asthmatic adults and healthy volunteers after the purpose of the test had been explained.

### Study subjects

A total of 52 healthy adult volunteers (24 women, 28 men; mean age 33.8±11.6 years) and 19 adult patients with asthma approximately matched for age (14 women, 5 men; mean age 33.0±12.3 years) were enrolled in this study. Asthmatic adults were dispensared at Allergy and Immunology outpatient clinics. Healthy volunteers had normal lung function test results. They had no clinical respiratory symptoms, and no history of acute respiratory infection during the last 4 weeks. They had no history of allergic, respiratory, cardiovascular, gastro-intestinal or endocrine diseases and were not treated with angiotensin-converting enzyme inhibitors (ACE inhibitors). Women included in the study were not pregnant.

Asthmatic adults in our study had mild asthma (according to the Global Initiative for Asthma – GINA update 2024) [19]. They did not experience daily asthma symptoms or their symptoms were quickly relieved. Only asthmatic adults without airway obstruction before inhalation cough challenge were enrolled in this study. Their Tiffeneau index (FEV1/FVC ratio) was equal to or more than 80 % of the predicted value, and their FEV1 was also equal to or more than 80 % of the predicted value (FEV_1_ – forced expiratory volume in 1 s; FVC – forced vital capacity), as reported in Table 1. Of the asthmatic adults, 57.9 % reported having a cough that occurred mainly during the day. Among them, 45.4 % had been experiencing a cough for more than three years. As many as 27.3 % reported cough lasting over two years, and another 27.3 % had cough that lasted more than one year. All asthmatic adults were non-smokers and had no history of acute respiratory infection during the last 4 weeks prior to the study. At the time of examination, they were in remission and were not using inhaled glucocorticoids or antihistamines.

### Research design

Each subject was asked about their history of asthma, as well as any allergic, cardiovascular, gastrointestinal, endocrine, or respiratory diseases and ACEI treatment. All subjects provided written informed consent before participation in the study. Healthy volunteers were examined in the laboratory, where bronchodilator treatment was available. Asthmatic adults were examined at an outpatient clinic, where first-aid medicaments were available. Pulmonary function testing was performed before and after the cough reflex sensitivity test. Spirometry was conducted using a KoKo DigiDoser-Spirometer; nSpire health Inc., Louisville, CO, USA. Each subject performed three forced expiratory maneuvers from total lung capacity to residual volume. FEV_1_ and FVC were recorded, and the best value for each was used in the analysis.

The assessment of cough reflex sensitivity to capsaicin (SIGMA, St. Louis, MO) was performed according to the ERS guidelines on the assessment of cough [14]. According to the guidelines, the single-breath dose-response method was preferred due to its accuracy, reproducibility of the dose delivered, and the ease of determination of tussive response. The cough reflex sensitivity was performed using a compressed air-driven nebulizer (KoKo DigiDoser; nSpire health Inc, Louisville, CO, USA) modified by the addition of an inspiratory flow regulator valve to 0.5 l.s^−1^ (RIFR; nSpire health Inc, Louisville, CO, USA) regardless of excessive inspiratory effort with each breath. Each subject inhaled a maximum of 12 prepared capsaicin aerosol concentrations (0.49–1000 μmol/l) for 1200 ms at 1 min intervals.

To increase cough challenge blindness, each subject inhaled physiological saline (control solution) randomly between incremental concentrations of capsaicin. This approach reduced the effect of voluntary suppression or conditioned responses in subjects who would otherwise expect progressively higher concentrations of capsaicin. By the single-breath method of capsaicin administration, each concentration of capsaicin was inhaled only once. Only coughs occurring within 15 s of capsaicin delivery were counted because the tussive response to each dose of aerosol is immediate and brief. The lowest concentrations of capsaicin required to reach two or more (C2) and five or more (C5) cough efforts were reported for each test. Subjects were instructed to cough if they needed and as much as they needed. They were unaware that achieving a specific number of coughs was the end-point of the study.

### Data analysis

Data for age and spirometric measurements were expressed as mean ± SD (standard deviation). Comparisons between FEV_1_ and FVC before and after the cough challenge were performed by using a paired *t-*test. A P<0.05 was regarded as statistically significant. The necessary sample size was calculated by G*Power software 3.1.9.7 for Windows. The values of cough reflex sensitivity were expressed as geometric mean values with a 95 % confidence interval (CI) of the capsaicin concentration causing two or more (C2) and five or more (C5) cough efforts for each group. Differences between groups were analyzed by non-parametric analysis of the Wilcoxon test with a statistically significant P value less than 0.05 (P<0.05).

## Results

The demographic data and spirometric measurements for adults who underwent capsaicin-cough challenge are shown in Table 1.

**Fig. 1:**
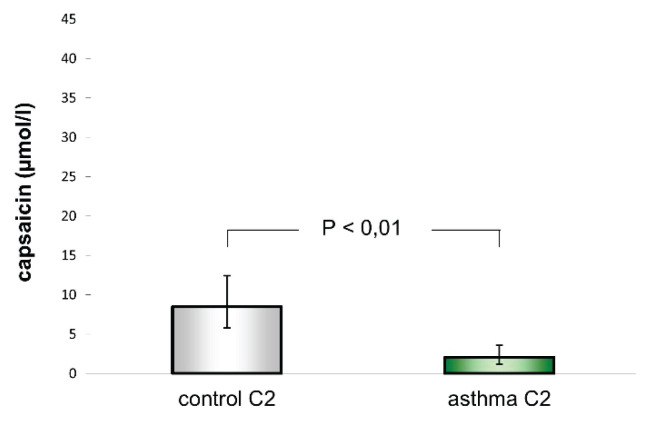
Cough reflex sensitivity evoking two or more coughs in healthy adult subjects (control C2) and asthmatic adult patients (asthma C2) – geometric mean and 95 % CI.

The capsaicin concentration evoking C2 was 2.03 μmol/ (1.16–3.55 μmol/l) in asthmatic adults, which was significantly lower than 8.46 μmol/l (5.76–12.41 μmol/l) in the control group of healthy adult volunteers (P<0.01). Cough reflex sensitivity expressed as C2 capsaicin concentration was significantly increased in adults with asthma (Fig. 1).

**Fig. 2:**
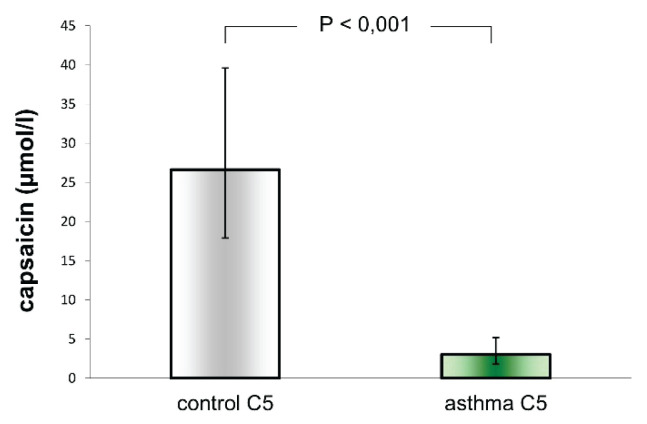
Cough reflex sensitivity evoking five or more coughs in healthy adult subjects (control C5) and asthmatic adult patients (asthma C5) – geometric mean and 95 % CI.

The concentration of capsaicin evoking C5 was 3.02 μmol/l (1.77–5.17 μmol/l) in asthmatic adults, compared to 26.62 μmol/l (17.90–39.58 μmol/l) in healthy volunteers. C5 capsaicin concentration in asthmatic adults was significantly lower than in the control group of healthy volunteers (P<0.001). Cough reflex sensitivity expressed as C5 capsaicin concentration was significantly increased in adults with asthma (Fig. 2).

Spirometric parameters FEV_1_, FVC and Tiffeneau index did not show any significant changes before and after cough reflex sensitivity test in both, asthmatic adults and healthy subjects (Table 1). We did not observe any serious adverse effects during testing. As many as 83.7 % of healthy subjects reported transient throat itching accompanying cough at higher concentrations of inhaled capsaicin and 6.5 % of healthy subjects reported retrosternal discomfort, such as a scratching or burning sensation. No other adverse effects were reported during the study.

## Discussion

The aim of this work was to determine and compare what is the threshold for inducing cough in adults with asthma and healthy adult volunteers, using the ERS recommendations. Few studies have investigated reference values for the capsaicin-cough provocation test in healthy adults, as reported by Koskela *et al*. [17] in their systematic review. They presented reference values for the single-breath capsaicin inhalation test based on data obtained by Prudon *et al*. [18]. However, they noted that these reference values are only applicable to results obtained using the same methodology. One of the main challenges in utilizing the obtained values of capsaicin concentrations to induce C2 and C5 in healthy adults as reference values for further clinical-physiological studies is the lack of standardization in cough challenge methodology used by different research groups. The methodology for the performance of inhalation cough challenge has been standardized to facilitate the universal interpretation and comparison of data generated by different laboratories [14]. In our previous clinical-physiological study, we analyzed two groups: 19 asthmatic patients and 38 healthy volunteers in the control group. We published preliminary results in the study by Neuschlova and Pecova [19]. To obtain more accurate data on C2 and C5 values, we have since expanded the control group to include 52 healthy adults. Several research groups have investigated the sensitivity of the cough reflex in adults, but the number of healthy volunteers in the control group was smaller than in our study. The only study that included a slightly larger group was conducted by Kanezaki *et al*. [20], which involved 56 healthy volunteers whose cough challenge was examined using capsaicin and the recommended ERS methodology. In subsequent studies involving adults, the control groups varied in size, consisting of 5 to a maximum of 40 healthy volunteers [21–28]. In the present clinical-physiological study, we confirmed our preliminary findings [19] that asthmatic patients demonstrate a significantly higher sensitivity of the cough reflex compared to healthy adults. Differences were observed in both C2 and C5. However, C5 showed a more significant difference (P<0.001) than C2 (P<0.01) in the present study. These results align with findings published by Dicpinigaitis *et al*. [29], which indicated that cough reflex sensitivity to capsaicin, measured as C5, serves as a sensitive indicator of changes in the reactivity of afferent nerve endings in the airways. The results obtained from our expanded group of healthy adult volunteers can be utilized as a control group for other clinical-physiological studies investigating the altered reactivity of afferent nerve endings in the airways in conditions such as chronic cough, asthma, chronic obstructive pulmonary disease, GERC, and many other diseases.

Our data showed that asthmatic adults started a cough response at significantly lower doses of capsaicin than healthy control subjects. This may indicate an increased excitability of the neural pathways involved in controlling cough in asthma. Several studies have shown similar results that asthma is associated with increased cough sensitivity to capsaicin inhalation, although different methodologies have been used to examine cough reflex. Satia *et al*. [10] reported that patients with stable asthma showed stronger cough responses to capsaicin suggesting possible neuronal dysfunction. Doherty *et al*. [30] found that subjects with asthma displayed greater sensitivity to capsaicin than healthy subjects. Furthermore, Nakajima *et al*. [31] demonstrated that patients with pure cough variant asthma had significantly higher cough sensitivity to capsaicin.

Positive findings on the cough provocation test are important in the diagnosis of atypical forms of asthma, such as CVA [32]. Although the cough provocation tests are not routinely performed in clinical practice, they enable evaluating the efficacy of therapy [22] and studying cough mechanisms. Several studies have used cough reflex sensitivity testing to clarify the main mechanisms leading to cough in patients with UACS [33], GERC [34,35], allergic rhinitis, and asthma [17,21,36] not only in adults but also in children with asthma [37,38], obesity and chronic cough [39].

Several studies have examined the relationship between the cough provocation test and respiratory function, but the results are contentious. In our study, the capsaicin inhalation challenge did not result in significant changes in spirometric parameters (FEV_1_, FVC, or FEV_1_/FVC) before and after the cough provocation test in healthy adults or adults with asthma. Fujimura *et al*. [40] reported that inhaled capsaicin significantly decreases FEV_1_ at the dose elicited 5 or more coughs in asthmatic patients. However, a more recent study by Chen [41], which performed a capsaicin inhalation challenge in healthy volunteers, patients with upper respiratory tract infection, patients with GERC, and asthmatic patients, found no significant differences in spirometric values before and after the capsaicin challenge test, which induced C5. Capsaicin does not induce clinically significant bronchoconstriction in healthy volunteers or asthmatic patients [14,42].

The cough provocation test is safe, well tolerated, and repeatable [41,43] and is useful in identifying patients with cough hypersensitivity, and quantitatively evaluating chronic cough. However, it cannot be used to assess cough frequency or severity [32].

The present study has some limitations. First, the study population of adults with asthma was much smaller than the control group of healthy volunteers. In the future, it will be necessary to expand the study groups of subjects to obtain more precise data, which will help clarify the functional changes in the airway afferent nerve endings mediating cough in asthma. Second, we focused on asthmatic adults with mild asthma who were well. Currently, it is unclear how to generalize these findings to groups with more severe forms of asthma. Third, we used only the univariate analyses of C2 and C5 parameters to compare study groups as they were central to the scope of this study.

In conclusion, we investigated capsaicin-evoked cough response in a group of adults with mild asthma and healthy adult volunteers as a control group using the standardized methodology recommended by ERS. We found increased reactivity of capsaicin-sensitive sensory nerves in asthmatic adults with mild stable disease. The data obtained can serve as baseline controls for future clinical-physiological studies comparing the reactivity of afferent nerve endings in the airways of patients with different asthma phenotypes and healthy volunteers. The cough reflex sensitivity measurement could provide valuable insights besides commonly used spirometry and inflammometric methods in clinical practice in asthma management.

## Figures and Tables

**Table 1:** Demographic and clinical characteristics of adults involved in the capsaicin-cough challenge.

*Characteristics of individuals*	Healthy adults (n=52)	Adults with asthma (n=19)
*Age (years)*	33.8 ± 11.6	33.0 ± 12.3
*Spirometry*		
* FEV* *_1_* * before challenge (%)*	104.0 ± 10.2	92.6 ± 8.9
* FEV* *_1_* * after challenge (%)*	100.9 ± 16.2	90.7 ± 10.2
* FVC before challenge (%)*	106.9 ± 9.7	91.5 ± 11.0
* FVC after challenge (%)*	106.6 ± 9.7	90.3 ± 12.5

Note: data are presented as mean ± SD (standard deviation). The data include age, parameters of forced expiration (FEV_1_ and FVC) before and after capsaicin-cough challenge. There were no statistically significant differences between spirometric parameters FEV_1_ and FVC before and after capsaicin-cough challenge in both adults with asthma and healthy adults.
